# Biological assessment and preservation strategies for SMILE- derived lenticules: towards a corneal regenerative biobank

**DOI:** 10.1038/s41598-026-50304-9

**Published:** 2026-04-29

**Authors:** Raimy Christodoulou, Domitilla Mandatori, Alessandro Boldini, Andrea Russo, Leonardo Mastropasqua, Mona El Zarif, Alessandro Ruzza, Letizia Pelusi, Assunta Pandolfi, Laura Pietrangelo, Stefano Ferrari, Diego Ponzin, Mario Nubile, Raluca Bievel Radulescu

**Affiliations:** 1 The Veneto Eye Bank Foundation, Venice, Italy; 2 Department of Medical, Oral and Biotechnological Science, Center for Advanced Studies and Technology-CAST, “G. d’Annunzio” University of Chieti-Pescara, Chieti, Italy; 3 Centro Oculistico Bresciano, Brescia, Italy; 4 Ophthalmology Clinic, Department of Medicine and Aging Science, “G. d’Annunzio” University of Chieti-Pescara, Chieti, Italy; 5 Genomic Surveillance and Biotherapy GSBT, Faculty of Sciences, Lebanese University, RasMaska, Lebanon; 6 Doctoral School of Sciences and Technology, Faculty of Public Health, Lebanese University, Hadath, Lebanon; 7 Department of Medicine and Aging Science, Center for Advanced Studies and Technology- CAST, “G. d’Annunzio” University of Chieti-Pescara, Chieti, Italy; 8 University of Ferrara, Ferrara, Italy; 9 Department of Translational Medicine, University of Ferrara, Ferrara, Italy

**Keywords:** KLEx, Stromal corneal lenticules, Tissue preservation, Lenticule biobank, Biotechnology, Diseases, Health care, Medical research

## Abstract

Human stromal corneal lenticules (hSCLs) extracted during keratorefractive lenticule extraction (KLEx) refractive surgery, including Small incision lenticule extraction (SMILE) procedure, are often discarded as medical waste, despite their potential for reimplantation in various ocular therapeutic applications. These include corneal thickness restoration in keratoconus, scleral patch grafts, and other ophthalmic procedures. Ensuring the safety and structural integrity of these lenticules for clinical use depends on proper preservation; however, a consensus on the most effective preservation method has yet to be reached. Hence, this study aimed to establish a comprehensive preservation protocol of SMILE-hSCLs, evaluating both pre-storage transportation media and long-term storage methods. In particular, we tested two pre-storage transport media, hyaluronic acid (HA) and Coldix, a dextran-containing MEM medium commonly used in our eye bank for corneal hypothermic storage, and three long-term (two weeks) preservation techniques represented by silica gel dehydration, cryopreservation in DMSO and glycerol. Our results demonstrated that hSCLs transported in either HA or Coldix for 48 h retained transparency, size, morphology, thickness and biological properties comparable to fresh tissues. However, hSCLs transported in HA exhibited superior overall collagen structural integrity. While, among the three tested long-term storage methods, both dehydration and cryopreservation in glycerol ensured effective preservation of corneal tissue. In summary, our findings identified HA as an effective pre-storage transport medium and demonstrated that both silica gel dehydration and glycerol-based cryopreservation are suitable strategies for the long-term preservation of SMILE-derived hSCLs. Although cryopreservation ensures good tissue preservation, the need for specialized equipment may pose a barrier to implementation in certain settings. In contrast, dehydration might offer a practical, low-cost solution ideal for establishing off-the-shelf hSCL biobanks, particularly in resource-limited settings. Collectively, these results lay the groundwork for a standardized and scalable preservation protocol to support future clinical use of KLEx-derived lenticules.

## Introduction

The global burden of corneal blindness remains a major public health challenge, ranking as the fourth leading cause of vision impairment worldwide^1^. Corneal transplantation is the standard therapeutic option for many corneal disorders, including keratoconus, stromal opacities, and scarring. However, the shortage of donor corneal tissue, especially in low-resource regions, severely limits access to timely and effective treatment^2^. This growing demand for corneal grafts underscores the urgent need for alternative sources of corneal tissue and innovative preservation strategies that can support tissue banking and distribution on a global scale. In recent years, keratorefractive lenticule extraction (KLEx) has emerged as an important class of refractive procedures for the correction of myopia and myopic astigmatism^3^. Among these, small incision lenticule extraction (SMILE), introduced following the development of femtosecond laser technology in 2008, has emerged as a widely adopted refractive surgery technique for the correction of myopia and myopic astigmatism^4,5^. Indeed, LASIK (laser-assisted in situ keratomileusis) has been the gold standard for vision correction surgery for decades, gaining widespread popularity since its approval in the 1990s^6^. However, while LASIK involves the formation of a corneal flap and the subsequent photoablation of the corneal stroma, in the KLEx procedure, a small incision is created to extract the stromal corneal lenticule (hSCL), thus eliminating the need for a corneal flap and making the procedure flapless and minimally invasive. During KLEx surgery, the extracted hSCL is generally discarded as medical waste. However, these lenticules represent a valuable source of viable stromal tissue with potential for reuse in a range of corneal and ocular surface reconstructive procedures. Indeed, the native collagen architecture, optical transparency, mechanical strength, and biocompatibility make hSCLs particularly highly suitable for tissue engineering and transplantation. Furthermore, hSCLs are autologous or allogeneic in origin and often derived from young, healthy donors, making them ideal for repurposing in ophthalmology^7^. Therefore, reusing hSCLs for ocular therapeutic applications has gained interest, particularly for corneal thickness restoration in keratoconus^8^, the creation of scleral patches^9–11^,and even as scaffolds for ocular drug release and cell-based regenerative therapies^12–14^.

Despite this promising potential, the clinical translation and widespread use of KLEx-derived hSCLs remain limited by the lack of standardized long-term preservation and storage protocols. Proper preservation is crucial not only for maintaining the structural and biological integrity of the tissue but also for ensuring safety, sterility, and long-term usability. In this regard, several methods have been proposed for long-term corneal tissue storage, including cryopreservation, dehydration, and hypothermic storage^15–19^. However, these techniques vary in effectiveness, cost, and logistical feasibility, and their suitability for hSCLs remains underexplored. In addition to this, pre-storage transport conditions are equally important, especially in scenarios where hSCLs must be shipped from surgical centers to biobanks or processing facilities. An optimal transport medium must protect the tissue, maintain hydration, transparency, and prevent structural degradation. In this context, the establishment of a corneal lenticule biobank built on validated transport and storage protocols could significantly enhance the availability of corneal tissue and support ocular regenerative therapies worldwide. Achieving this goal requires the identification of an optimal storage protocol for hSCLs through a comprehensive understanding of both short-term transport conditions and long-term preservation strategies.

To address this critical gap, the present study aims to develop a standardized protocol for KLEx-derived hSCLs storage, encompassing both the optimal pre-storage transport medium and the most effective long-term preservation method to ensure safety, along with the preservation of biological and structural integrity. Specifically, we tested two transport media, hyaluronic acid (HA) and Coldix, a hypothermic corneal storage medium commonly used in our eye bank, alongside three long-term preservation strategies applied over two weeks: silica gel dehydration, cryopreservation in DMSO, and cryopreservation in glycerol. These approaches were selected based on their cost-effectiveness, practicality, and their potential to preserve stromal tissue architecture. Our overall goal is to establish a ready-to-use biobank of KLEx-derived hSCLs to address the global shortage of corneal tissue and SMILE lenticules for therapeutic applications. Such a biobank would provide a valuable and readily available resource for ophthalmic purposes, offering stored stromal lenticules for a wide range of therapeutic applications, including keratoconus treatment and scleral reinforcement, thereby enhancing access to corneal tissue for reimplantation.

## Materials and methods

### Ethical statement

This study was conducted in accordance with the Declaration of Helsinki and was approved by the Ethical Committee for Clinical Trials (CESC) of the Province of Venice (Italy) and the IRCCS San Camillo (262 A/CESC/ approved 18.04.2023). Informed consent was obtained from all subjects and hSCLs were collected from Centro Oculistico Bresciano, Brescia, Italy, and Centro Nazionale di Alta Tecnologia in Oftalmologia, Chieti, Italy. No organs or tissues were procured from prisoners for this study.

### Donor selection

Donor selection followed strict criteria in accordance with the requirements set by the national competent authority, the Italian National Transplant Service (Centro Nazionale Trapianti, Rome, Italy). hSCLs were obtained from healthy living donors who underwent SMILE refractive surgery using the VisuMax system (Carl Zeiss Meditec, Germany) and met predefined inclusion and exclusion criteria. Eligible subjects were informed about the study during their anamnesis visit and provided signed informed consent. Inclusion criteria included age 18–70, myopic or astigmatic refractive defects, and suitability for SMILE surgery. Exclusion criteria included ocular conditions such as ocular hypertension, keratitis, keratoconus, corneal dystrophies, and other intrinsic eye diseases. Donors were also screened for active infections, including bacterial, viral, fungal, or parasitic diseases. Specifically, individuals with active cytomegalovirus, West Nile virus, infectious endocarditis, myocarditis, SARS, tuberculosis, meningitis, syphilis, malaria, leprosy, or borreliosis were excluded. Risk factors for HIV, HBV, or HCV, such as intravenous drug use, high-risk sexual behaviours, recent incarceration, or non-sterile tattoos/piercings, also disqualified donors. Neurological disorders, including Creutzfeldt-Jakob disease, dementia, encephalitis, Parkinson’s, Alzheimer’s, multiple sclerosis, ALS, and Guillain-Barré syndrome were exclusionary. Additional criteria included hematopoietic diseases (e.g., leukaemia, lymphoma), recent vaccinations with live attenuated viruses, malignant neoplasms, and genetic conditions like Marfan, Noonan, and Down syndromes.

### hSCLs collection and storage conditions

**Fig. 1:**
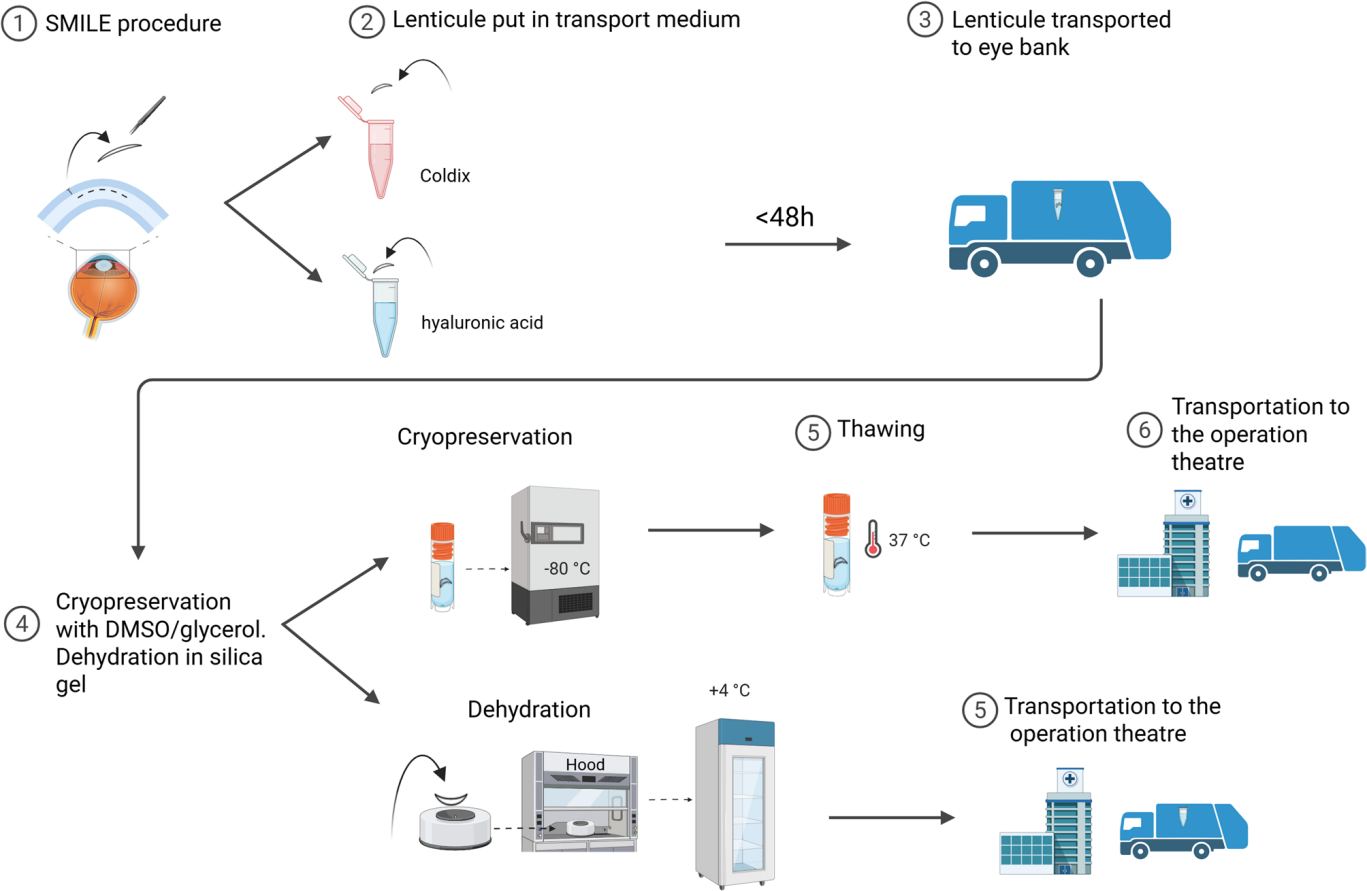
Graphical overview of the experimental workflow. A total of 117 hSCLs were collected at two clinical centers: Centro Oculistico Bresciano, Brescia, Italy and Centro Nazionale di Alta Tecnologia in Oftalmologia, Chieti, Italy. SMILE-derived hSCLs were placed in HA or in Coldix. Both were transported within 48 h to Fondazione Banca degli Occhi (FBOV). Upon arrival, 69 HA or Coldix transported hSCLs were immediately analyzed. The remaining 48 hSCLs were then preserved using three different long-term storage conditions: dehydration in silica gel, cryopreservation with DMSO or cryopreservation in glycerol. For the long-term storage samples were stored for two weeks before analyses. Figure created with BioRender.com (https://www.biorender.com)*.*

A total of 117 hSCLs from 78 donors were obtained from Centro Oculistico Bresciano (Brescia, Italy) and Centro Nazionale di Alta Tecnologia in Oftalmologia (Chieti, Italy). As reported in the experimental workflow in Fig. 1, hSCLs were placed in one of two pre-storage transport media: HA (IAL-F, 1.8% sodium hyaluronate, Bausch & Lomb) or Coldix (a dextran-containing MEM-based hypothermic storage medium produced in-house according to EU Regulation 2017/745 [MDR]). When available from the same donor, one lenticule was stored in HA and the contralateral lenticule in Coldix. All hSCLs, regardless of the pre-storage medium, were transported within 48 h to the Fondazione Banca degli Occhi (FBOV). Upon arrival at the eye bank, hSCLs were rinsed in sterile phosphate-buffered saline (PBS) to remove any residual transport medium. Subsequently, 69 hSCLs were analyzed to assess the effects of 48-hour transport in HA or Coldix.

The remaining 48 hSCLs were used to evaluate three following long-term preservation techniques:

*Dehydration in Silica gel*: hSCLs (n = 16) were placed on a white silicone base and left to dry under a Class A laminar flow hood for 1.5 h. They were then stored in a sterile 35 mm Petri dish in a container with sterile silica gel at 2–4 °C for 14 days. Following storage, hSCLs were rehydrated in sterile PBS for 15 min before analyses.

*Cryopreservation in DMSO*: hSCLs (n = 16) were placed into cryovials containing 1 mL of a DMSO cryopreservation solution composed of 80% base medium (Base, Alchimia srl, Ophthalmic Surgery and Human Tissue Processing, Padova, Italy), 10% dimethyl sulfoxide (DMSO), and 10% human albumin (Alburex, CSL Behring GmbH, Marburg, Germany). The cryovials containing the hSCLs and preservation medium were then placed into Mr. Frosty and kept at -80 °C for 14 days.

*Cryopreservation in Glycerol*: hSCLs (n = 16) were placed into cryovials containing 1 mL of glycerol without additional supplements and stored at -80 °C for 14 days.

### Diameter and thickness measurements

The diameter and thickness of the SMILE-derived hSCLs were measured using Optical Coherence Tomography (Casia, AS-OCT, Tomey, Japan). hSCLs were placed in a 35 mm diameter Petri dish and the centre of each lenticule was identified. The central lenticule thickness (CLT) of each hSCL was determined by averaging three measurements, each within a range of ± 2 μm. The diameter was calculated by centring the lenticule and measuring its edge-to-edge distance.

### Transparency measurements

Transparency was measured using a custom-built device by checking the light intensity with a probe, as previously described by Parekh et al.20. Measurements were obtained using a pinhole to ensure data accuracy. Each hSCL was placed in a Petri dish with a 35 mm diameter. All measurements were aligned using a blank Petri dish as a reference. For each sample, three measurements were taken, and the average was calculated.

### Histology

For histological analyses, SMILE-derived hSCLs were fixed in 4% paraformaldehyde and embedded in paraffin. Paraffin blocks were then cut into 5 μm thick slices. Four different stains were performed using an automatic stainer (LEICA autostainer XL; Leica Biosystems, Nussloch, Germany): Hematoxylin and Eosin (H&E; Bio-Optica, Milano, Italy) Masson’s trichrome (MT; Bio-Optica, Milano, Italy), Periodic Acid-Schiff (PAS; Kaltek, Saonara, Italy) and Alcian Blue (AB; Diapath SpA, Martinengo, Italy). Quantification analyses of MT, PAS and AB were performed by using ImageJ software (NIH, United States ImageJ software, public domain available at:"http://rsb.info.nih.gov/nih-image/).

### Transmission electron microscopy (TEM)

hSCLs were processed for Transmission Electron Microscopy (TEM) morphology evaluation and morphometric analysis. Samples were fixed with 3.5% glutaraldehyde in 0.1 M sodium cacodylate (NaCaCO) buffer for 1 h and stored at 4 °C. For embedding, hSCLs were fixed in 2% OsO4 in the same buffer for 2 h and block-stained in uranyl acetate replacement. Ultrathin sections (~ 50 nm) were cut using a Leica Ultracut R microtome (Leica Microsystem; Weltzar, Germany) with a Diatome diamond knife (Diatome Ltd., Nidau Switzerland) and double-stained with uranyl acetate replacement and lead citrate. Sections were viewed at 8 kV in a JEM 1400 Flash TEM (Jeol, Tokyo, Japan) equipped with a sCMOS “Matataki” camera and Sight X Viewer Software Ver.2.1.26.1818. For the quantitative analyses in each sample, cross-sectional views of hSCLs were imaged as follows: collagen fibrils were evaluated at a magnification of 12-14.000X in 3 random fields from 3 different control and decellularized hSCLs. In each micrograph, the collagen fibril density parameter was evaluated and expressed as number/µm2, and the mean fibril size was measured and reported in nm2. The structure of collagen fibers was evaluated at a magnification of 4.000X. Collagen fibers were classified into highly organized, partially organized, and disorganized fibers by the presence of a precise disposition of fibrils inside each fiber/lamella or a partial/complete disorganization. In detail, highly organized collagen lamellae were identified by long collagen fibrils, which run parallel to one another with the same orientation; partially organized fibers were composed of collagen fibrils with intermediate levels of ultrastructural organisation (e.g. shorter and/or not regularly spread); finally, disorganized fibers presented irregularly spread collagen fibrils, often fragmented in the long axis - leaving collagen-free regions - and/or presenting different orientations. The number of highly organized, partially organized, and disorganized fibers present in each sample was counted and reported as a percentage of the total fibers.

### Statistical analyses

Descriptive statistics, including mean and standard deviation, were calculated for all groups across measurements of thickness, transparency, diameter, collagen fibril density and fibers organization. To compare only the HA and Coldix groups, the Mann-Whitney test was performed. For comparisons among more than two groups, Ordinary one-way ANOVA or Kruskal-Wallis followed by Tukey’s or Dunn’s post hoc test was used to determine significant differences. The significance level was set at alpha = 0.05 (95% confidence intervals). All statistical analyses were conducted using GraphPad Prism 10 software (GraphPad, San Diego, CA, USA).

## Results

### Effect of pre-storage transport media on hSCLs

**Fig. 2:**
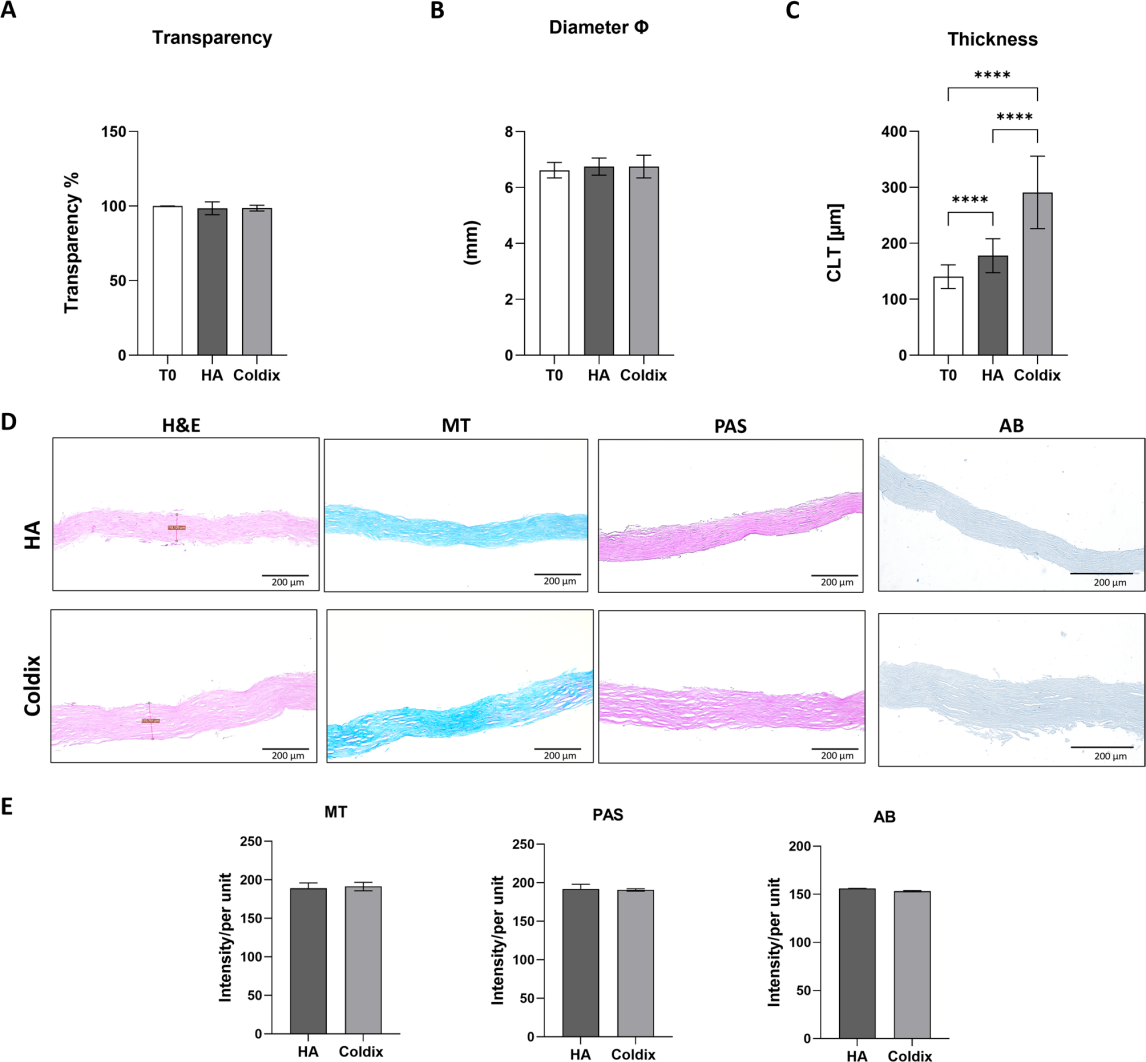
Effect of Pre-Storage Transport Media on hSCLs. (**A**) Transparency of hSCLs before (T0; fresh control) and after transportation in either HA or Coldix. (**B**) Diameter of hSCLs before (T0; fresh control) and after transportation in either HA or Coldix. (**C**) Central thickness of hSCLs before (T0; fresh control) and after transportation in either HA or Coldix. (**D**) Representative histological images: Hematoxylin and Eosin (H&E), Masson’s Trichrome (MT), Periodic Acid-Schiff (PAS) and Alcian Blue (AB). (**E**) Quantification analyses of MT (collagen), PAS (glycoproteins) and AB (glycosaminoglycans) staining intensity. Results are presented as a mean ± standard deviation (SD). Total number of hSCLs analyzed: HA (*n* = 32) and Coldix (*n* = 31). Statistical analyses were performed using Ordinary one-way ANOVA with Tukeys’s *post hoc* test and the Mann-Whitney test. ****p < 0.0001.

**Fig. 3:**
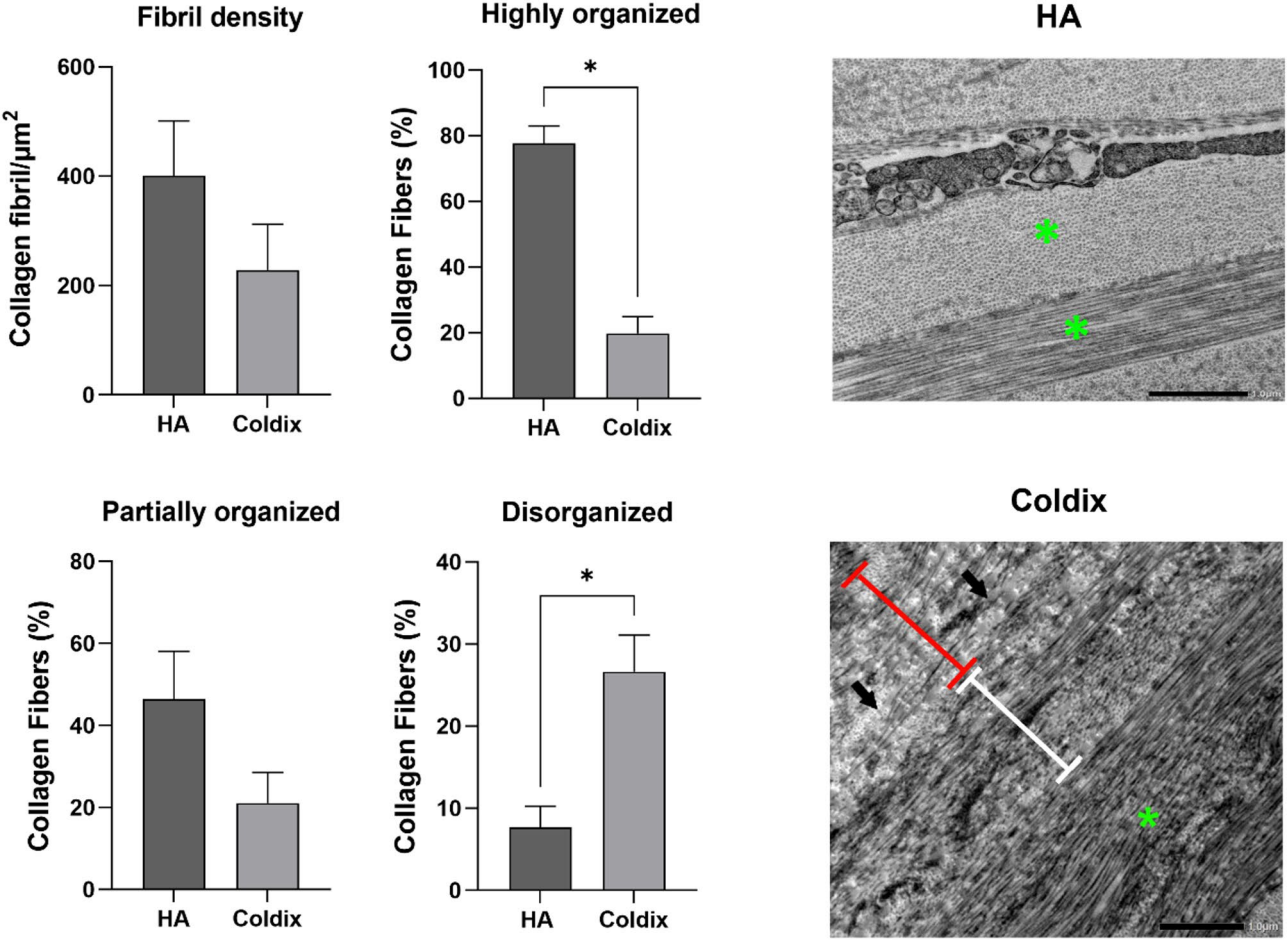
Ultrastructural Effect of Pre-Storage Transport Media on hSCLs. Morphometric and representative images of TEM analyses showing collagen fibril density and different fibers organization in HA and Coldix transported hSCLs. Total number of hSCLs analyzed: HA (*n* = 3) and Coldix (*n* = 3). Results are presented as a mean ± standard deviation (SD). Statistical analyses were performed using the Mann-Whitney (test). *p < 0.05. Scale bar: 1 μm. Green asterisks mark highly organized fibers; white bar identify a partially organized fiber; red bar marks a disorganized fiber with collagen free regions (black arrows).

To establish a reference standard for preserved hSCLs, we first analyzed the characteristics of 69 extracted hSCLs transported for 48 h in either HA or Coldix. We show that transparency, a key quality indicator for potential clinical applications, remained consistently high during transportation in HA or Coldix, with an average of 98% transparency in both groups compared to the fresh control (baseline measurement performed immediately after hSCLs extraction; T0 baseline 100%) (Fig. 2A). Furthermore, our findings indicate that also the diameter of hSCLs remains stable at 6.6 mm before (at T0) and after transportation in both HA and Coldix (Fig. 2B), suggesting that neither transport solution affects lenticule size. Although the initial CLT value measured in fresh hSCLs was comparable between groups (around 140 μm), a highly significant increase in CLT was observed in hSCLs transported in Coldix compared to those transported in HA. Indeed, hSCLs transported in HA exhibited an increase of 37.1 μm compared to fresh control (T0), whereas those transported in Coldix showed a dramatic increase of 150.9 μm (Fig. 2C). In addition, histological analyses performed using H&E revealed structural differences between the two pre-storage transportation conditions. More in detail, hSCLs transported in Coldix exhibited a slightly looser and less cohesive stromal architecture, with visible gaps between collagen fibers. In contrast, those transported in HA showed a more preserved stromal structure, with collagen fibers appearing more continuous, aligned, and better maintained, closely resembling the physiological organization observed in fresh tissue (Fig. 2D). However, as revealed by MT, PAS and AB quantification analyses, the content of collagen, glycoproteins and glycosaminoglycans in the stromal extracellular matrix of hSCLs appeared comparable between the HA and Coldix transport conditions, suggesting that transport did not significantly alter the overall composition of the hSCLs stromal matrix (Fig. 2E). The hSCLs structural alteration was further analysed by TEM analyses (Fig. 3), which confirmed that transportation in HA preserves the ultrastructural organization of collagen fibrils and fibers. In contrast, samples transported in Coldix exhibited a significant reduction in highly organized fibers, accompanied by a marked increase in disorganized fibers, suggesting that while both media support hSCLs integrity during transportation, HA may offer superior preservation of stromal organization compared to Coldix.

### Long-term preservation methods

After assessing the effects of HA and Coldix as pre-storage transport media, we proceeded to evaluate the three long-term preservation methods: silica gel dehydration, cryopreservation in DMSO or glycerol.

#### Characterization of hSCLs following silica gel dehydration long-term storage

**Fig. 4:**
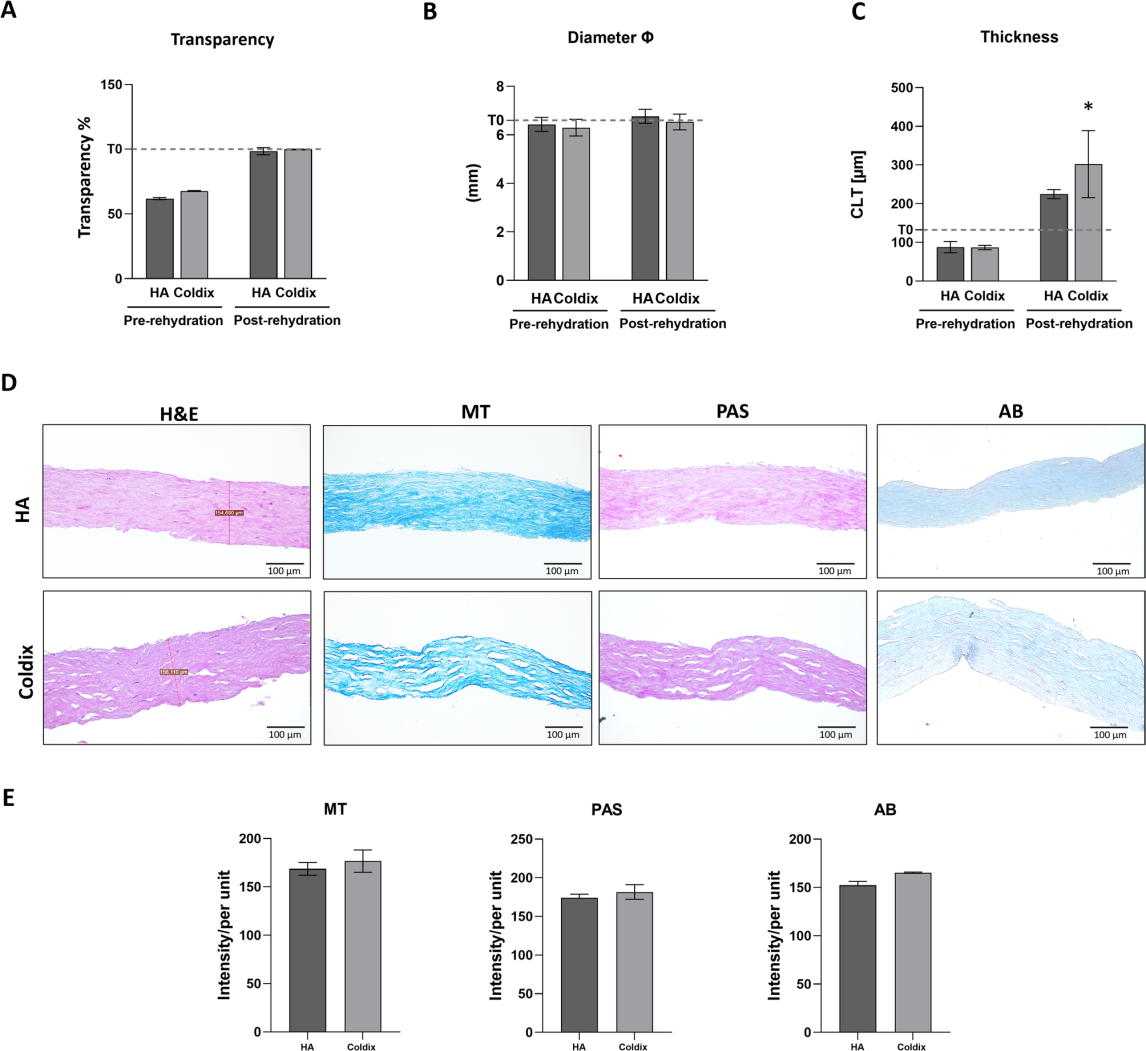
Characterization of hSCLs following Silica Gel Dehydration Long-Term Storage. (**A**) Transparency of hSCLs, transported in either HA or Coldix, before and after rehydration following storage in silica gel. (**B**) Diameter of hSCLs, transported in either HA or Coldix, before and after rehydration following storage in silica gel. (**C**) Central lenticule thickness (CLT) of hSCLs, transported in either HA or Coldix, before and after rehydration following storage in a dehydrated state. (**D**) Representative histological images: Hematoxylin and Eosin (H&E), Masson’s Trichrome (MT), Periodic Acid-Schiff (PAS) and Alcian Blue (AB). (**E**) Quantification analysis of MT (collagen), PAS (glycoproteins) and AB (glycosaminoglycans) staining intensity. Results are presented as mean ± standard deviation (SD). Total number of hSCLs analyzed for dehydration: HA (*n* = 3) and Coldix (*n* = 3), for a total of *n* = 6. Statistical analyses were performed using Kruskal-Wallis with Dunn’s *post hoc* test. *p < 0.05 versus T0 baseline (fresh control; measurements performed before hSCLs transport in HA or Coldix).

The first tested method was dehydration in silica gel for 14 days. As shown in Fig. 4A, transparency remained consistently high in post-dehydration, with both groups exhibiting 99.9% transparency compared to T0 baseline line (100%; measurements performed before transport in HA or Coldix), suggesting a limited impact of the dehydration process on optical clarity. In addition, dehydration did not significantly affect the diameter of hSCLs in either group, as both pre- and post-dehydration measurements averaged 6.6 mm (Fig. 4B). Regarding the hSCLs thickness, following the dehydration in silica gel (pre-rehydratation condition), the CLT value decreased to 87.6 μm in the HA group and 86.6 μm in the Coldix group. After 15 min of rehydration in PBS, the thickness increased to 224.6 μm and 302.3 μm, respectively. While the increase observed in the HA group was not statistically significant compared to the fresh control (T0 baseline line thickness: 143.6 μm), the increase in the Coldix group was statistically significant (Fig. 4C). Furthermore, histological examination (Fig. 4D) demonstrated a notable difference between the two groups: hSCLs originally transported in HA exhibited a more cohesive and less disrupted structure, with fewer gaps between collagen fibers and a higher degree of alignment. In contrast, hSCLs transported in Coldix appeared looser, more fragmented, with less uniform collagen fibers organization. However, the content of collagen, glycoproteins and glycosaminoglycans also appeared preserved among the HA and Coldix group, as confirmed by the quantitative histochemical analyses (Fig. 4E).

#### Characterization of hSCLs following cryopreservation long-term storage

**Fig. 5:**
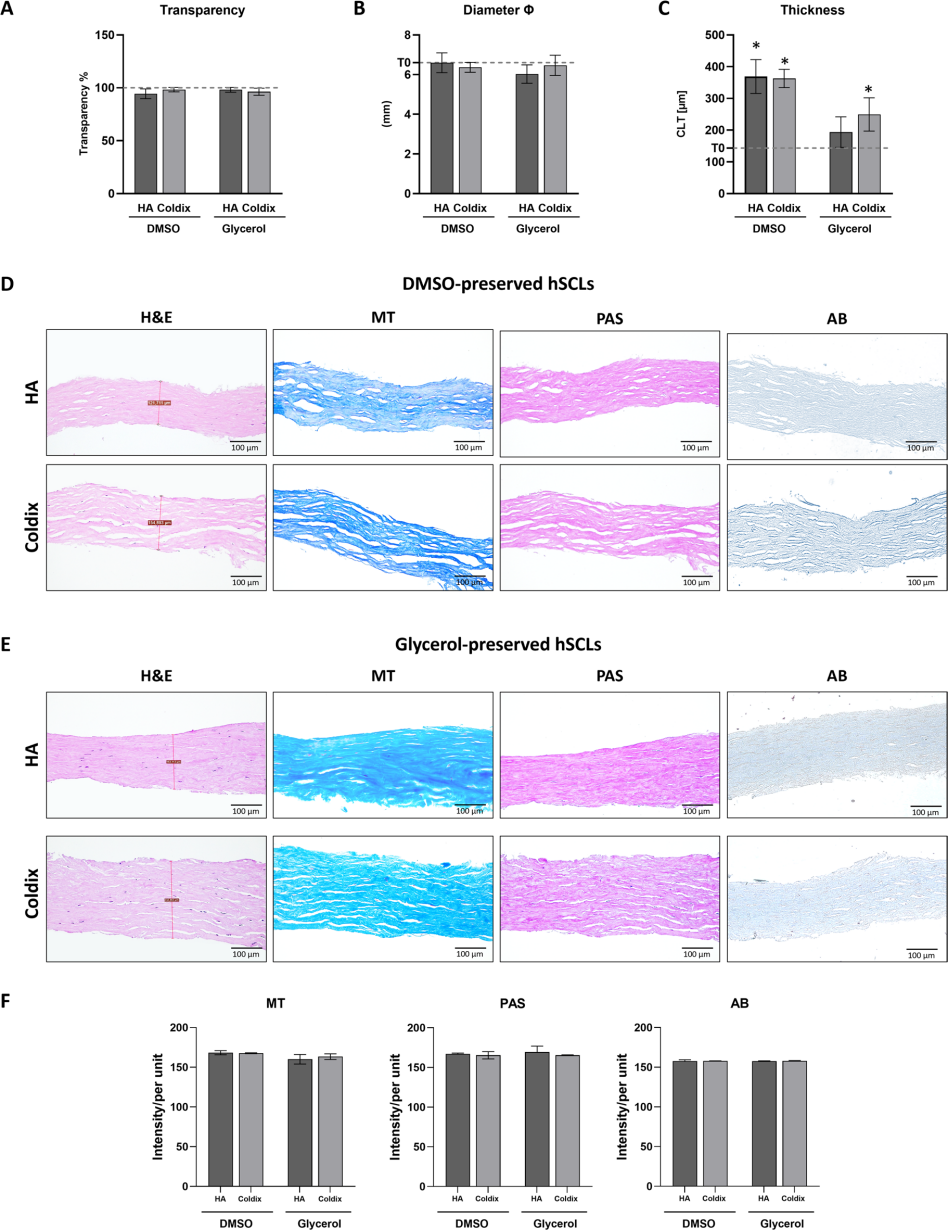
Characterization of DMSO or Glycerol Cryopreserved hSCLs. (**A**) Transparency of hSCLs, transported in either HA or Coldix, after cryopreservation in DMSO or glycerol. (**B**) Diameter of hSCLs, transported in either HA or Coldix, after cryopreservation in DMSO or glycerol. (**C**) Central lenticule thickness (CLT) of hSCLs, transported in either HA or Coldix, after cryopreservation in DMSO or glycerol. (**D**,** E**) Representative histological images of DMSO- and glycerol-preserved hSCLs: Hematoxylin and Eosin (H&E), Masson’s Trichrome (MT), Periodic Acid-Schiff (PAS) and Alcian Blue (AB). (**F**) Quantification analysis of MT (collagen), PAS (glycoproteins) and AB (glycosaminoglycans) staining intensity of DMSO and glycerol-preserved hSCLs. Number of hSCLs analyzed: HA/DMSO (*n* = 3), HA/Glycerol (*n* = 3), Coldix/DMSO (*n* = 3), and Coldix/Glycerol (*n* = 3). Total: *n* = 6 for DMSO cryopreservation and *n* = 6 for glycerol cryopreservation. Results are presented as mean ± standard deviation (SD). Statistical analyses were performed using Kruskal-Wallis with Dunn’s *post hoc* test. *p < 0.05 versus T0 baseline (measurements performed before transport in HA or Coldix).

Similar to dehydration, cryopreservation did not significantly affect hSCLs transparency in either group (Fig. 5A). Likewise, cryopreservation in DMSO or glycerol had no significant impact on diameter, which remained unchanged (Fig. 5B).

In contrast, cryopreservation in DMSO led to a significant increase in hSCL thickness in both HA and Coldix pre-storage groups compared to the fresh control baseline value measured at T0 (before transport in HA or Coldix; 143.6 μm). In detail, hSCLs transported in HA showed an average thickness increase of 226 μm, reaching 369 μm post-cryopreservation, while those transported in Coldix increased by 219 μm, reaching an average thickness of 362 μm (Fig. 5C). Similarly, cryopreservation in glycerol resulted in an increase in thickness in both groups compared to the CTL value measured at T0 (fresh control). More in detail, hSCLs transported in HA exhibited a thickness increase of 50 μm, reaching an average post-cryopreservation thickness of 193 μm. In contrast, hSCLs transported in Coldix showed more than double the thickness increase (106 μm), reaching an average thickness of 249.3 μm. Notably, the increase observed in the HA group was not statistically significant compared to the T0, while the increase in the Coldix group was significant, suggesting that cryopreservation in glycerol after HA transport may better preserve hSCLs thickness and structure.

This result was further supported by histological analyses, which revealed structural differences between the two cryopreservation methods (Fig. 5D, E). In particular, hSCLs preserved in DMSO (Fig. 5D), exhibited a disorganized, porous, and disrupted collagen structure in both HA and Coldix pre-storage groups, whereas glycerol-preserved hSCLs exhibited a more intact and organized structure with cohesive and aligned collagen fibers (Fig. 5E). Despite these structural differences, no differences were observed in the content of extracellular matrix proteins, like collagen, glycoproteins and glycosaminoglycans (Fig. 5F).

#### Effects of long-term storage on hSCLs collagen ultrastructure

**Fig. 6:**
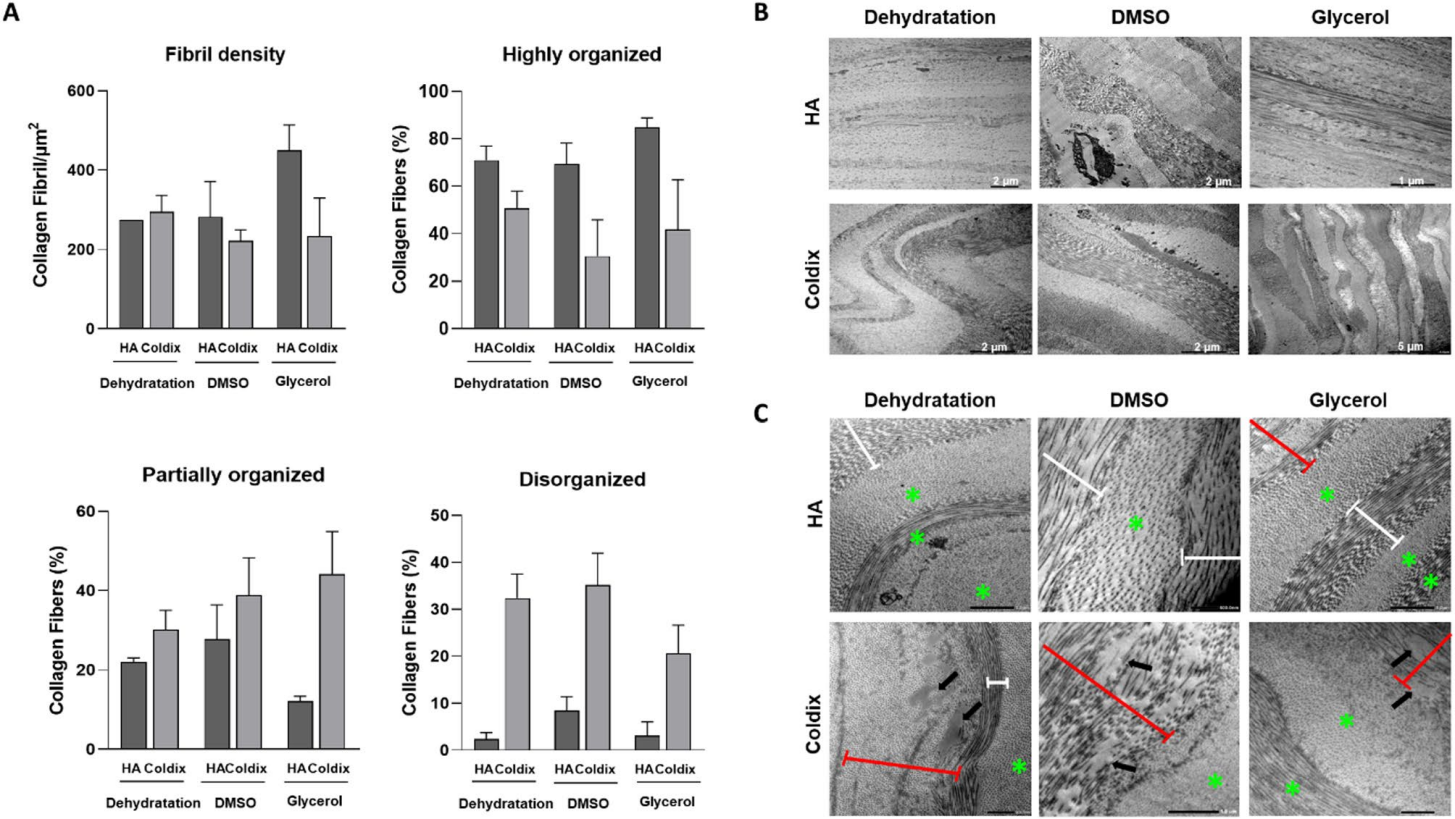
Morphometric and representative images of transmission electron microscopy (TEM). (**A**) Analyses showing collagen fibril density and different fibers organization in hSCLs transported in HA or Coldix, followed by preservation through silica gel dehydration or cryopreservation in DMSO or glycerol. Results are presented as a mean standard deviation (SD). (**B**) Lower and (**C**) higher magnification TEM representative images showing collagen fibril density and fibers organization in hSCLs transported in HA or Coldix for 48 h, and followed by preservation through silica gel dehydration or cryopreservation in DMSO or glycerol. Green asterisks mark highly organized fibers; white bar identify a partially organized fiber; red bar marks a disorganized fiber with collagen free regions (black arrows). Number of hSCLs analyzed: HA/Dehydration (*n* = 3), HA/DMSO (*n* = 3), HA/Glycerol (*n* = 3), Coldix/Dehydration (*n* = 3), Coldix/DMSO (*n* = 3), and Coldix/Glycerol (*n* = 3), for a total of *n* = 18. Statistical analyses were performed using Kruskal-Wallis with Dunn’s *post hoc* test.

Quantitative and qualitative analyses of hSCLs collagen ultrastructure by TEM (Fig. 6) revealed notable differences in structural preservation depending on the specific combination of transport medium and long-term storage method employed. First, we confirmed that HA transport provides superior preservation of hSCLs stromal ultrastructure compared to Coldix, as evidenced by the increased levels of partially organized and disorganized fibers in samples transported in Coldix. In contrast, hSCLs pre-stored in HA showed consistently higher collagen fibril density and a greater proportion of highly organized fibers. These effects were more noticeable in hSCLs that were subsequently dehydrated in silica gel or cryopreserved in glycerol for long-term storage (14 days). Indeed, among the three preservation techniques tested (silica gel dehydration, cryopreservation in DMSO or glycerol), both dehydration and glycerol cryopreservation showed a superior ability to preserve collagen ultrastructure of hSCLs, maintaining a stromal organization that closely resembles the physiological architecture of fresh lenticules. Specifically, transporting hSCLs in HA, followed by either silica gel dehydration or glycerol-based cryopreservation, resulted in increased fibril density, improved collagen fibers organization, and a reduction in disorganized fibers, identifying these combinations as the most effective strategies for preserving collagen structure over the long term.

### Discussion

Corneal blindness continues to rank among the leading causes of vision loss worldwide. One of the major challenges in addressing this condition is the limited availability of donor tissue, which restricts access to corneal transplantation, especially in resource-limited settings. In this context, hSCLs extracted during KLEx refractive surgery have gained attention as a novel and promising source of autologous or allogeneic stromal tissue for corneal repair and regenerative therapies^7^.

The concept of reusing KLEx-derived hSCLs was first validated in animal models by Angunawela et al., who demonstrated that implanted hSCLs could effectively restore stromal volume^21^. Soon after, Pradhan et al. translated this concept into clinical practice by implanting an allogeneic lenticule in a young patient to treat hyperopia, showing that this strategy could serve as a solution for correcting high refractive errors^22^. Since then, multiple studies have confirmed the therapeutic potential of hSCLs implantation to improve corneal shape, biomechanical stability, and visual function in conditions such as keratoconus and post-LASIK ectasia^8–14^. However, most of the lenticules used in these studies were freshly harvested and transplanted within hours or days, a logistical limitation that restricts broader clinical implementation. Therefore, to fully harness the potential of KLEx-derived hSCLs, there is a pressing need to develop a reliable biobanking system supported by validated transport and long-term preservation protocols. This would not only enable separating the timing of donation from transplantation but also ensure the availability of ready-to-use lenticules for both elective and emergency surgical interventions. In this context, although evidence on the preservation of hSCLs is still emerging^23^, early studies suggest that cryopreservation can maintain structural integrity and collagen organization^15–19^. Noriega et al. in 2011 demonstrated that stromal lenticules extracted from refractive surgery remain viable after cryopreservation^17^, while Ganesh et al. reported the first successful implantation of a glycerol-cryopreserved SMILE-derived lenticule in 2014 for the treatment of hyperopia^24^, marking a critical step toward the use of stored lenticules.

Although these findings highlight the potential of cryopreservation for lenticule banking, additional evidence supporting the clinical feasibility of preserved corneal tissues has also emerged from studies on dehydrated grafts. Several dehydration techniques have been proposed for stromal tissue preservation, including air-drying under a laminar flow hood followed by storage in silica gel, as performed in this study, as well as controlled dehydration under low pressure and mild heat^25,26^. Moreover, clinical outcomes have shown that dehydrated corneal stromal tissues perform comparably to fresh organ-cultured donor corneas in deep anterior lamellar keratoplasty (DALK)^27^, suggesting that dehydration may represent a valid alternative preservation strategy for stromal tissues. Nevertheless, further studies are needed to determine the maximum safe storage duration for dehydrated lenticules.

In addition to the mentioned cryopreserved and dehydration-based approaches, alternative preservation strategies have also been recently explored. Among these are hydrogel nutrient capsules, a relatively recent method designed to mimic the natural tear film environment through capsules composed of natural polysaccharides. In this context, previous studies have reported that this system can maintain lenticule transparency, collagen organization, and cellular viability for up to one year^28,29^. Furthermore, a subsequent clinical study in which capsule-preserved lenticules were transplanted into ten patients reported no complications, with good tissue transparency and integration observed one year after surgery^30^. However, despite these promising findings, there is currently no consensus on the optimal preservation method that ensures both safety and the retention of key biological and structural features of hSCLs^31^. Therefore, our study aimed to fill this gap by identifying and standardizing a storage method that best supports the structural and biological integrity of KLEx-derived hSCLs, while also being practical and scalable for clinical application and biobanking.

Since the choice of transport medium could significantly influence lenticule integrity before long-term preservation, we compared the hypothermic solutions known as Coldix, a dextran-containing MEM-based solution produced in-house for corneal preservation, with the use of HA as an alternative transport medium. The rationale behind this choice is that HA is a naturally occurring glycosaminoglycan widely used in ophthalmology since the 1980s for its viscoelastic and lubricating properties^32^. Importantly, HA also exhibits superior water retention and high zero shear viscosity compared with other polymers^33^ which prompted us to investigate its potential as an alternative transport medium for hSCLs. However, despite HA’s widespread use in ophthalmology, to our knowledge, there are no prior reports evaluating its efficacy for KLEx derived-hSCLs transport, making this the first study to compare its effects with Coldix.

Interestingly, our results demonstrate that hSCLs transported in HA present a stromal architecture closer to physiological conditions compared to Coldix. While HA maintains tissue hydration, Coldix appears to induce osmotic stress, which compromises collagen fiber structure. Indeed, consistent with the structural characteristics of fresh KLEx-derived lenticules described in the literature^14,34,35^, hSCLs transported in HA exhibited higher collagen fibril density and a more organized fibers arrangement, whereas those transported in Coldix showed increased disruption and disorganization of the stromal matrix. Furthermore, a key finding of our study was that hSCLs transported in Coldix showed a marked increase in thickness, whereas those transported in HA exhibited only a mild change. This difference can be attributed to HA’s superior water retention properties and higher viscosity, which likely limit excessive swelling compared to Coldix, which is essentially an aqueous solution. Accordingly, these findings indicate that Coldix has a more pronounced effect on hSCLs hydration and swelling than HA, a consideration that may influence its suitability for lenticule preservation and therapeutic use. However, despite significant thickness differences, diameter and transparency remained consistently high in both transport medium groups, with only minimal variation compared to fresh control (T0 values), indicating that short-term transport does not significantly compromise lenticule size and its key optical property. We propose two possible explanations for these observations. First, unlike the naturally curved architecture of the cornea, the relatively flat configuration of the lenticule may limit the impact of thickness changes on collagen fibers alignment, thereby preserving both size and transparency. In addition, a homogeneous distribution of the absorbed fluid within the stromal matrix may help maintain the spatial arrangement of collagen fibrils, thus preventing significant alterations in light transmission^36,37^. Secondly, it could be that our transparency measuring device may not have been sensitive enough to detect subtle differences. While reproducible and suitable for routine eye-bank evaluation, it may lack the sensitivity to detect subtle thickness-related differences in light scatter. Previous validation studies of this instrument indicated that it provides an overall measure of transparency but does not resolve small or layer-specific changes^20^. Importantly, the findings related to thickness, diameter and transparency are best understood in relation to the T0 baseline measurements obtained immediately after lenticule extraction (fresh control). Indeed, these structural and optical parameters were assessed at T0 and subsequently re-evaluated after their transport in HA or Coldix, enabling a direct longitudinal comparison within the same lenticules. By contrast, stromal structure was evaluated through histological and TEM analyses performed only after the hSCLs transport to assess the effects of the different conditions. A direct T0 histological and TEM control was not included, as this would have precluded longitudinal evaluation of the same hSCL before and after transport. Although this aspect represents a limitation of the study, as it does not allow a direct within-sample comparison of ultrastructural features at baseline, the structural characteristics of freshly extracted lenticules have been well described both in the literature and in our previous work^14,17,29,34,35^, consistently demonstrating a compact stromal architecture characterized by highly organized collagen fibers and regular fibril arrangement. In this context, the alterations observed following transport, particularly in the Coldix group, suggest a partial loss of the native stromal organization, whereas lenticules transported in HA tend to present structural features more closely resembling physiological stromal architecture of fresh lenticule. Therefore, the collagen architecture and the limited increase in thickness position HA as a more suitable transport medium. This is particularly critical, as the preservation process begins immediately after transport, and any structural compromise at this early stage could negatively influence the effectiveness of long-term storage and clinical outcomes. Once transported, KLEx-derived hSCLs must be stored for days to months while retaining their physical and biological properties. In terms of long-term preservation, we evaluated three different approaches: dehydration in silica gel, cryopreservation either in glycerol or DMSO^24,38,39^.

Dehydration has long been used to store corneas because it reduces water content and suppresses enzymatic activity^16^. Glycerol is a viscous, hygroscopic cryoprotectant with antimicrobial properties. It removes water, reduces ice crystal formation during freezing, and allows corneas to be stored for years without losing clarity and stromal architecture^40^. Our findings are consistent with previous studies^26,39^, indicating that both dehydration and cryopreservation in glycerol are effective options for hSCLs conservation. By contrast, cryopreservation in DMSO, though widely used for cell storage, proved less favourable for stromal architecture. DMSO is an effective cryoprotectant because it readily penetrates tissues and prevents ice crystal formation, and although it effectively preserves keratocyte viability, it has a negative impact on collagen fibers^41,42^. Indeed, one common limitation of both glycerol cryopreservation and dehydration, however, is the loss of viable stromal keratocytes. Nevertheless, this is generally not considered a major concern, as previous studies have shown that host-derived keratocytes gradually repopulate the lenticule after transplantation^39^. Additionally, given DMSO’s chemical properties, it could potentially interact with proteoglycans and glycosaminoglycan in the extracellular matrix, which could further explain the loss of cohesiveness and increased porosity. In contrast, glycerol, a slow-penetrating cryoprotectant, better stabilizes collagen structure and prevents abrupt swelling^43^. This makes it a more suitable option for long-term storage of KLEx-derived lenticules. Although glycerol cryopreservation appears to be the method that best preserves the collagen ultrastructure of hSCLs, dehydration should not be overlooked. In our study, silica gel dehydration-maintained transparency and collagen organization nearly as well as glycerol cryopreservation. These findings differ from those reported by Xia et al., who directly compared lenticule preservation in glycerol and silica gel over a four-week period. Similar to our observations, collagen fibril organization remained relatively regular in both groups, however a reduction in fibril density was reported. Moreover, unlike our results, lenticules preserved in silica gel maintained transparency comparable to fresh controls, whereas those stored in glycerol exhibited reduced transparency^44^. While, in our experimental setting, hSCLs preserved either by silica gel dehydration or glycerol cryopreservation maintained transparency levels comparable to fresh control (T0 value). We also found that, although an increase in thickness relative to T0 value was observed in both groups, this did not result in a loss of lenticule stromal organization. Nevertheless, it should be noted that, also for the long-term storage experiments, a direct comparison with T0 was not available for histological and TEM analyses, as baseline processing would have precluded longitudinal evaluation of the same lenticule. Despite this limitation, the increase in thickness from both silica gel dehydration and glycerol cryopreservation did not appear to compromise the overall stromal architecture, which remained compact and qualitatively comparable to that of fresh lenticules^14^.

From a practical perspective, however, it is important to note that dehydration has significant logistical advantages. Indeed, cryopreservation requires expensive equipment that may not be available in all clinical settings worldwide. Although Long-term glycerol cryopreservation offers extended shelf life, it may require − 80 °C freezers to maximize efficacy, whereas dehydrated hSCLs can be stored at room temperature or in standard refrigerators and rehydrated when needed. This makes dehydration an attractive method for developing off-the-shelf hSCLs that can be easily stored and shipped, making it particularly beneficial for resource-limited areas with poor infrastructure. In light of these advantages, a tiered preservation strategy could be envisioned for the development of an KLEx- derived hSCLs biobanks: dehydration could be used for readily accessible hSCLs intended for emergency interventions, while glycerol-based cryopreservation may serve as the preferred method for long-term biobanking in well-equipped facilities. Therefore, the combination of structural preservation achieved through HA transport and long-storage *via* glycerol or dehydration is likely sufficient to support a wide range of therapeutic applications. These include use as patch grafts for corneal perforations, lacerations, and stromal scarring. Moreover, preserved hSCLs could be employed in elective procedures, such as intrastromal implants for keratoconus or corneal augmentation in eyes with thin stroma. In this context, it is also important to consider the potential of hyperopic KLEx, which yields negative lenticules particularly suitable for replenishing stromal tissue and restoring corneal thickness in keratoconus^45,46^. Additionally, with continued progress in laser reshaping and tissue engineering techniques, including decellularization, recellularization and bioengineering process^12,14^, KLEx-derived hSCLs may be precisely modified and reshaped according to specific clinical needs, further expanding their therapeutic potential for personalized and regenerative corneal application. Nonetheless, establishing a dedicated biobank of KLEx-derived hSCLs requires regulatory approval, standardized protocols, and quality control. Therefore, a regulatory approval from the National Transplant Center of Italy will be required to enable the clinical distribution of preserved hSCLs. In this context, despite the encouraging results obtained in this study, several limitations should be acknowledged. The aim of this work was to test and establish a standardized protocol for both the transportation and storage of KLEx-derived hSCLs, identifying an optimal transport medium and evaluating feasible preservation strategies. For this reason, the study was designed as a *proof-of-concept* investigation focusing on the methodological setup of the transport and storage workflow. However, the long-term preservation protocols were evaluated for a relatively short storage period of 14 days. While this timeframe allows an initial comparative assessment of the different preservation strategies, it does not fully reflect the longer storage durations that would be required in a real biobanking *scenario*. In a translational perspective, lenticules collected during KLEx refractive surgery may need to be preserved for substantially longer periods, potentially ranging from several months to up to one year before clinical use. Therefore, future studies will be necessary to evaluate the stability, structural and optical integrity of lenticules over extended storage intervals, including additional checkpoints such as 1, 3, 6, and 12 months. In addition, as previously discussed, although lenticules parameters like transparency, diameters and thickness were longitudinally compared with the fresh control (baseline T0 values), histological and TEM morphometric analyses were not performed at T0, both for the short-term transport and long-term storage conditions. This represents a limitation of the study, as it prevents a direct comparison of ultrastructural features with the native hSCLs baseline structure. Therefore, future studies will therefore include T0 histological and ultrastructural analyses to enable a more comprehensive evaluation of structural changes over time. Finally, another limitation of the present study is the relatively limited sample size used. Hence, increasing the number of samples in future investigations will allow a more robust statistical assessment and further validate the reproducibility of the preservation strategies proposed in this study. In this regard, future studies involving larger cohorts and potentially including additional specialized centers involved in KLEx-derived lenticule collection will be particularly important to strengthen the robustness and generalizability of the proposed protocols. Overall, these considerations help to better frame the significance of our results and highlight how the proposed transport and storage strategies may support future studies aimed at developing standardized protocols for lenticule storage. Ultimately, the development of validated preservation protocols could enable the establishment of dedicated biobanks of KLEx-derived hSCLs, which may significantly increase the availability of corneal graft material and expand therapeutic options for corneal repair and regeneration.

## Conclusions

Our data support the feasibility of using HA as a transport medium and either glycerol cryopreservation or dehydration for long-storage. The combination of HA transport and glycerol cryopreservation offers the most robust preservation of collagen ultrastructure and is therefore recommended for long-term banking. Dehydration provides a cost-effective alternative for settings lacking cryopreservation infrastructure. Implementing these strategies can enable eye banks to create readily available, structurally sound lenticules, reducing dependence on donor corneas and expanding access to corneal regenerative therapies.

## Data Availability

All data supporting the findings of this study are included within the article.
